# Intermittent fasting for the prevention of cardiovascular disease: implications for clinical practice

**DOI:** 10.12968/bjca.2023.0058

**Published:** 2023-09-26

**Authors:** O Hamer, A Abouzaid, J Hill

**Affiliations:** University of Central Lancashire; NHS Blackpool Teaching Hospitals NHS Foundation Trust; University of Central Lancashire

**Keywords:** Systematic review, Cardiovascular diseases, Intermittent fasting, Diet, Blood pressure, Epidemiology

## Abstract

Cardiovascular disease remains one of the most prevalent and preventable chronic conditions worldwide. Nutrition plays an important role in reducing several risk factors associated with cardiovascular disease. Intermittent fasting has been rapidly gaining interest among patients with cardiometabolic disease as a nutritional strategy for improving cardiovascular outcomes. However, research had yet to determine whether intermittent fasting provides greater cardiometabolic benefits compared to continuous daily caloric restriction. A recent Cochrane review has synthesised the benefits of intermittent fasting for the prevention of cardiovascular disease but is limited by its interpretation of the findings for clinical practice. This commentary aims to critically appraise the methods used within the review by Allaf et al, 2021 and expand upon the findings to determine its implications for clinical practice.

## Introduction

Cardiovascular disease remains one of the most prevalent and preventable chronic conditions worldwide (1). Data suggests that cardiovascular disease (CVD) is likely to be the primary cause of mortality in Western society, accounting for over seventeen million annual deaths (2, 3). Nutrition plays an important role in reducing the risk factors associated with cardiovascular disease, some of which include heightened blood pressure, excess weight, inflammation, and increased cholesterol (4–7). The risk of cardiovascular disease (CVD) can be influenced by several aspects of nutrition, including calorie intake, dietary composition, and meal timing (8, 9). Recent literature has highlighted that regulating the timing of meals to avoid night-time hours (such as during intermittent fasting) may decrease risk factors associated with cardiovascular disease (e.g., adiposity) (10, 11).

Intermittent fasting (IF) has been rapidly gaining interest among the general population and patients with cardiometabolic disease (e.g., adults with obesity) (12). The term intermittent fasting is often defined as a reduced caloric intake on an intermittent basis which can vary from several hours during the day to a complete 24-h period (e.g., 5:2, TRE, ADF and Modified ADF) (1). Unlike conventional calorie restriction, IF allows individuals to achieve caloric reduction while maintaining regular eating patterns, presenting a more sustainable dietary approach for long-term adherence (13). Intermittent fasting has several prosed mechanisms, the most recognised being a belief that it may lower calorie intake, leading to a metabolic shift that enhances fat metabolism and reduces fat stores (14).

Intermittent fasting approaches such as the 5:2 diet, and time-restricted eating (TRE) have been found to produce mild to moderate weight loss (1-8% from baseline), and consistent reductions in energy intake (10-30% from baseline) (15). IF may also benefit cardiometabolic health by decreasing blood pressure, insulin resistance, low-density lipoprotein cholesterol, triglyceride levels and oxidative stress (16). However, findings related to these outcomes are inconsistent, ranging between no effect to moderate effectiveness (14, 16–19). Although some evidence favours intermittent fasting, it is still unclear whether it provides additional cardiometabolic benefits as compared to routine care of continuous daily caloric restriction (19). Some preliminary data suggest that IF regimens may provide cardiometabolic benefits in the absence of weight loss, however most studies to date have found little to no evidence to support this premise (18, 19). With a range of conflicting evidence, a recent Cochrane systematic review aimed to provide an up-to-date summary of the benefits of IF for the prevention of cardiovascular disease (17). This commentary aims to critically appraise the methods used within the review by Allaf et al, 2021 and expand upon the findings to specifically = determine its implications for clinical practice (17).

## Methods Of The Cochrane Review By Allaf Et Al. (2021)

A comprehensive multi-database search was undertaken from date of inception to December 2019 (including the Cochrane Central Register of Controlled Trials, Embase and MEDLINE). Additional trial registries were searched for eligible studies. Further to this, reference lists of included studies were screened for relevant studies. Only random controlled trials which assessed the effect of intermittent fasting compared to either non-restrictive feeding or continuous energy restriction in adults (with or without cardiac risk) were included. Cross over trials and RCT’s that didn’t meet a minimum 12-hour caloric restriction criterion (25% or less of maintenance caloric requirement) were excluded. Religious fasts, such as Christian Lent fasts, Daniel Fasts, Buddhist fasts, and Jewish fasts, that didn’t meet this criterion were also excluded.

Screening, data extraction and assessment of bias (Risk of bias assessment tool, RoB1) was undertaken independently by a minimum of two reviewers (20). The certainty of the effect estimates was assessed using the Grading of Recommendations, Assessment, Development, and Evaluations approach (GRADE) (21). A fixed effects (I^2^<50%) or a random effects model (I^2^>50%) was used when conducting meta-analysis to generate a mean estimation of effect (where outcome data was available). Where applicable, a range of sensitivity and subgroup analyses were performed on several moderating factors (subtypes of intermittent fasting, females only versus non-females only, overweight, and obese only versus non-overweight only, and diabetes only versus non-diabetes only).

## Results Of The Cochrane Review By Allaf Et al. (2021)

Out of 39,165 records identified by the electronic database searches, 26 studies were included of which 18 studies provided sufficient data for meta-analysis. Notably, all 26 studies were deemed to be high risk of bias due to a lack of blinding of participants, allocation concealment, selective reporting, or incomplete outcome data (i.e., attrition bias).

### Comparison 1 - Intermittent fasting (IF) compared to ad libitum feeding (short term, eating at any time with no specific caloric restriction) for the prevention of cardiovascular disease

There was a statistical but nonclinical significant short-term (≤ 3 months) reduction in body weight (kg), BMI (kgm^2^), waist circumference, total cholesterol levels and systolic blood pressure compared to ad libitum (eating at any time with no specific caloric restriction) (see table 1 for statistics).

There was no evidence of difference in the short-term (≤ 3 months) for low-density lipoprotein cholesterol levels, high-density lipoprotein cholesterol levels, total triglyceride levels, diastolic blood pressure, C-reactive protein and fasting plasma glucose levels in adults who underwent intermittent fasting compared to those who ate at any time with no specific caloric restriction (ad libitum) (see table 1 for statistics).

### Adverse events for all studies

A total of four trials reported on side effects. Pooled data showed that 13 out of 187 participants in the intermittent fasting groups experienced headaches (7%).

### Comparison 2 – Intermittent fasting (IF) compared to short term continuous energy restriction (CER) for the prevention of cardiovascular disease

When compared to continuous energy restriction there was a statistical but not clinically significant small reduction in body weight (kg) and BMI (kgm^2^) compared to short term CER (see table 2 for statistics).

There was no evidence of difference in waist circumference, total cholesterol levels, low-density lipoprotein cholesterol levels, high-density lipoprotein cholesterol levels, total triglyceride levels, systolic blood pressure, diastolic blood pressure, C-reactive protein and fasting plasma glucose levels in adults who underwent intermittent fasting compared to those who underwent short term CER (ad libitum) (see table 2 for statistics).

### Comparison 3 - Intermittent fasting (IF) compared to medium term continuous energy restriction (CER) for the prevention of cardiovascular disease

There was no evidence of difference in the medium term (> 3 months to 12 months) for body weight (kg), BMI (kgm^2^), waist circumference (cm), total cholesterol levels, low-density lipoprotein cholesterol levels, high-density lipoprotein cholesterol levels, total triglyceride levels, systolic blood pressure, diastolic blood pressure, C-reactive protein and fasting plasma glucose levels in adults who underwent intermittent fasting compared to those who underwent medium term CER (see table 3 for statistics).

## Commentary

By employing the AMSTAR-2 critical appraisal tool for systematic reviews, all 16 criteria were met as indicated in Table 4 (22). Consequently, the systematic review was deemed to be an accurate and comprehensive overview of existing evidence. It is important to note that the authors of the review decided to only grade specific outcomes with no clear rationale why all outcomes examined were not assessed using the grade criteria. This does not impact on the quality of the review but does make it more difficult to interpret the evidence in context to practice.

The Cochrane review highlighted that intermittent fasting could have favourable benefits for body weight, BMI, waist circumference, total cholesterol levels and systolic blood pressure (compared to ad libitum feeding) (17). However, although the benefits were statistically significant, they were not deemed clinically significant according to established cut off values for each outcome (e.g., a blood pressure reduction of 5mmHg, weight loss of 5% etc) (17, 23). Notably, the reduction for waist circumference was at the borderline of clinical cut off but this was based upon data from only two studies. Additionally, the findings identified that little is currently known of the benefits of intermittent fasting compared to ad libitum feeding in the mid-to long-term (> 3 months) (17).

### Implications for practice

The findings of this review were established based on four types of intermittent fasting: alternate day fasting (ADF), modified alternate day fasting (Modified ADF), periodic fasting (PF) and time-restricted feeding (TRE) (17). These intermittent fasting types have several commonalities which include cyclical feeding patterns (alternating between periods of fasting and periods of ad libitum), caloric restriction during fasting periods, ad libitum feeding during non-fasting periods, and specific fasting schedules (e.g., alternate day fasting) (24, 25). The review highlighted that intermittent fasting may produce mild to moderate weight loss over short durations (i.e.., 8–12 weeks), however, the ability of intermittent fasting to help to manage weight over a longer term is still unknown (17). In addition, the quality of evidence supporting these findings was low to very low which restricts the application of intermittent fasting as a means to achieve weight loss or sustained weight loss in an adult population (17). Further high quality RCT’s are needed to confirm the effectiveness of intermittent fasting on weight related outcomes in both the short and longer term.

The findings of the review also highlighted that there is a dearth of literature which has reported the potential adverse events of intermittent fasting. Where adverse events were reported, data suggests that approximately 7% of participants experienced mild to severe headaches (n = 13/187) (17). This finding is consistent with recent evidence highlighting that adults undergoing IF interventions occasionally suffer mild headaches, fatigue, and constipation in the first few weeks, but that these generally resolve after three weeks (26, 27). Although recent literature has deemed intermittent fasting as largely safe (with few gastrointestinal, neurological, hormonal or metabolic adverse effects), practitioners should be cautious about encouraging its adoption given the dearth of literature surrounding its safety profile (15). At a minimum, patients considering intermittent fasting (without professional guidance) should be informed of the potential transient effects (likely experienced in the first few weeks) to facilitate awareness and potential reduce unnecessary visits to health services (28). Further high quality RCT’s are needed to assess the safety and efficacy of intermittent fasting.

Further to its unclear safety profile, the review findings identified high attrition rates among those undertaking intermittent fasting interventions (approx. 15%) (17). This is consistent with the scope of recent research which has reported attrition rates as high as 26% within IF interventions (29). High attrition rates can be problematic for several reasons, including patient acceptability and tolerability, misrepresentation of intervention effectiveness and concerns of bias (28, 30). The attrition rates found in the current review suggest that practitioners may find it challenges to implement the dietary intervention within clinical practice (17). Patients may terminate the intervention because they find it difficult to sustain and this could impact on its wider feasible for the target population (29, 31). In addition to concerns related to patient tolerability, high attrition rates could lead to a misrepresentation of intervention effectiveness through overestimation or underestimation of effect (30). If participants who withdraw experience adverse effects or negative outcomes, intermittent fasting may appear more effective from the absence of their data. Furthermore, when attrition rates are high, the remaining sample may not represent the target population, compromising the generalizability of the findings (32). Further research in the form of a qualitative study may be needed to establish the important reasons why patients abandon intermittent fasting and in which contexts withdrawal may be more frequent. Furthermore, an intention-to-treat analysis should be carried out in all future studies.

Due to the low certainty of evidence and high-risk of bias within existing studies, recommendation for intermittent fasting to be adopted into clinical practice cannot yet be made. At present, there is several limitations that need addressing before recommendations to practise can be made which include a lack of safety data, the absence of high quality RCT’s (with low risk of bias), a dearth of clinically significant findings compared to usual care (e.g., calorie restriction diets), and the absence of valid risk-benefit analysis for specific patient populations (e.g., individuals with obesity or diabetes).

### Implications for future research

Further high quality RCT’s are required to assess the effectiveness of intermittent fasting on weight related outcomes (e.g., BMI and body weight), blood pressure, cardiometabolic outcomes, all-cause mortality, cardiovascular mortality, stroke, myocardial infarction, heart failure and cholesterol. Further high quality RCT’s are also needed to assess the safety of intermittent fasting, its efficacy, and provide a valid risk-benefit analysis for different patient groups. In relation to this, an RCT comparing intermittent fasting, calorie restriction, and unrestricted eating over a greater time-period (>24 months) is needed to evaluate adverse events. High-quality long-term studies (>24 months) are also needed (with regular follow-up), to explore the potential benefits of intermittent fasting on the outcomes mentioned above. These trials should include a range of patients with and without established cardiovascular disease, as well as those with cardiovascular risk factors.

### CPD reflective questions

- What are the strengths of the systematic review by Allaf et al?
- What advice can be given to patients about intermittent fasting?
- What are the key limitations of the systematic review discussed in this commentary and what needs to be considered before we can make recommendations for application to clinical practice?

## Figures and Tables

**Table 1:** Intermittent fasting (IF) compared to short term ad libitum feeding (eating at any time with no specific caloric restriction) for the prevention of cardiovascular disease.

Outcome	Relative effect / mean difference (95% CI)	No. of studies	Heterogeneity I^2^ statistic	GRADE
**Body weight**	MD -2.88 kg, (95% CI -3.96 to -1.80)	7	85%	Low
**BMI**	MD of -0.92 kg/m^2^ (95% CI -1.36 to -0.48)	4	61%	NR
**Waist circumference**	MD -4.19 cm, (95% CI -6.38 to -2.01)	2	0%	NR
**Total cholesterol levels**	MD -0.31 mmol/L, (95% CI -0.51 to -0.12)	4	0%	NR
**LDL cholesterol levels**	MD -0.22 mmol/L, (95% CI -0.40 to 0.05)	4	0%	NR
**HDL cholesterol levels**	MD -0.10 mmol/L, (95% CI -0.25 to 0.05)	4	65%	NR
**Total triglyceride levels**	MD -0.06 mmol/L, (95% CI-0.25 to 0.14)	4	50%	NR
**SBP**	MD -4.47 mmHg, (95% CI -6.94 to -2.01)	5	0%	NR
**DBP**	MD -1.07 mmHg, (95% CI -3.33 to 1.18)	5	0%	NR
**C-reactive protein** **(CRP) (mg/L)**	MD -1.19 mg/L, (95% CI-2.54 to 0.16)	2	0%	NR
**Glucose and glycated haemoglobin (HbA1c)**	MD -0.03 mmol/L, (95% CI -0.26 to 0.19)	3	15%	Very low
**Side effects/adverse events**	NR	0	NR	NR
**Side effects/adverse events**	13 out of 187 participants in the intermittent fasting groups had at least a mild headache (7%: 4 studies).	4	NR	NR

* MD = Mean difference, NR = Not reported

**Table 2:** Intermittent fasting compared to short term continuous energy restriction (CER) for the prevention of cardiovascular disease.

Outcome	Relative effect / mean difference (95% CI)	No. of studies	Heterogeneity I^2^ statistic	GRADE
**Body weight**	MD -0.88 kg, (95% CI -1.76 to 0.00)	10	66%	Very low
**BMI**	MD -0.43 kg/m^2^, (95% CI -0.76, to -0.10)	9	34%	NR
**Waist circumference**	MD -0.83 cm, (95% CI -2.11 to 0.44)	8	60%	NR
**Total cholesterol levels**	MD -0.07 mmol/L, (95% CI -0.18 to 0.03)	8	0%	NR
**LDL cholesterol levels**	MD -0.07 mmol/L, (95% CI -0.16 to 0.01)	9	0%	NR
**HDL cholesterol levels**	MD -0.01 mmol/L, (95% CI -0.06 to 0.04)	9	59%	NR
**Total triglyceride levels**	MD -0.07 mmol/L, (95% CI -0.19 to 0.06)	8	43%	NR
**SBP**	MD -1.75 mmHg, (95% CI -4.61 to 1.11)	7	24%	NR
**DBP**	MD -0.97 mmHg, (95% CI -2.35 to 0.42)	7	0%	NR
**C-reactive protein (CRP) (mg/L)**	MD 0.31 mg/L, (95% CI -0.56 to 1.17)	2	0%	NR
**Glucose and glycated haemoglobin (HbA1c)**	MD -0.02 mmol/L, (95% CI -0.16 to 0.12)	9	73%	Very low

* MD = Mean difference, NR = Not reported

**Table 3:** Intermittent fasting compared to medium term continuous energy restriction (CER) for the prevention of cardiovascular disease.

Outcome	Relative effect / mean difference (95% CI)	No. of studies	Heterogeneity I^2^ statistic	GRADE
**Body weight**	MD -0.56 kg, (95% CI -1.68 to 0.56)	4	0%	Low
**BMI**	MD -0.15 kg/m^2^, (95% CI -0.58 to 0.29)	4	0%	NR
**Waist circumference**	MD -0.66 cm, (95% CI -2.55 to 1.23)	3	58%	NR
**Total cholesterol levels**	MD -0.04 mmol/L, (95% CI -0.17 to 0.10)	3	0%	NR
**LDL cholesterol levels**	MD -0.06 mmol/L. (95% CI -0.18 to 0.05)	3	0%	NR
**HDL cholesterol levels**	MD -0.00 mmol/L, (95% CI -0.07 to 0.07)	3	52%	NR
**Total triglyceride levels**	MD -0.02 mmol/L, (95% CI -0.16 to 0.12)	4	0%	NR
**SBP**	MD 1.37 mmHg, (95% CI -4.98 to 7.72)	3	52%	NR
**DBP**	MD -1.00 mmHg, (95% CI -4.67 to 2.67)	3	37%	NR
**C-reactive protein (CRP) (mg/L)**	MD 0.46 mg/L, (95% CI -0.87 to 1.79)	1	NR	NR
**Glucose and glycated haemoglobin (HbA1c)**	MD 0.01 mmol/L, (95% CI -0.10 to 0.11)	4	0%	Low

* MD = Mean difference, NR = Not reported

**Table 4:** Critical appraisal using the AMSTAR-2 tool for assessing systematic reviews

AMSTAR 2 items	Responses
1. Did the research questions and inclusion criteria for the review include the components of PICO?	Yes – The study outlined the PICO’s in the methods section.
2. Did the report of the review contain an explicit statement that the review methods were established prior to the conduct of the review and did the report justify any significant deviations from the protocol?	Yes – The protocol was registered on the Cochrane Database of Systematic Reviews.
3. Did the review authors explain their selection of the study designs for inclusion in the review?	Yes - Studies included RCT’s in which the participants underwent intermittent fasting compared to ad libitum feeding (normal diet) or caloric restriction for the primary or secondary prevention of cardiovascular disease.
4. Did the review authors use a comprehensive literature search strategy?	Yes - Electronic searches of three databases and several conference proceedings were included.
5. Did the review authors perform the study selection in duplicate?	Yes – Study selection was independently conducted by five reviewers.
6. Did the review authors perform data extraction in duplicate?	Yes - Data extraction was conducted by two reviewers who spot checked the other’s extracted data.
7. Did the review authors provide a list of excluded studies and justify the exclusions?	Yes - The authors provided reasons for exclusion and listed the studies in a table.
8. Did the review authors describe the included studies in adequate details?	Yes – A characteristics of included studies table was available.
9. Did the review authors use a satisfactory technique for assessing the risk of bias in the individual studies that were included in the review?	Yes - Two reviewers independently assessed the methodological quality of included studies.
10. Did the review authors report on the sources of funding for the studies included in the review?	Yes – The authors reported funding sources for each included study where the data was available.
11. If meta-analysis was performed did the review authors use appropriate methods for statistical combination of results?	Yes - Meta-analysis was conducted with appropriate methods using a random effects models.
12. If meta-analysis was performed did the review authors assess the potential impact of RoB in individual studies on the results of the meta-analysis or other evidence synthesis?	Yes - The study conducted a sensitivity analysis to assess the potential impact of bias in individual studies on the results.
13. Did the review authors account for RoB in individual studies when interpreting/discussing the results of the review?	Yes – The authors considered the risk of bias of included studies when discussing the results
14. Did the review authors provide a satisfactory explanation for and discussion of, any heterogeneity observed in the results of the review?	Yes – The authors explored heterogeneity within each outcome.
15. If they performed quantitative synthesis did the review authors carry out an adequate investigation of publication bias (small study bias) and discuss its likely impact on the results of the review?	Yes – the review conducted a GRADE assessment which included an assessment of publication bias.
16. Did the review authors report any potential sources of conflict of interest, including any funding they received for conducting the review?	Yes - The authors reported no competing or conflicting interests.

* PICO = Participants, Intervention, Comparator & Outcome.

## Key Points

1. Intermittent fasting may reduce body weight, BMI, waist circumference, total cholesterol levels and systolic blood pressure compared to eating at any time with no specific caloric restriction (ad libitum) in the short term.
2. Intermittent fasting may slightly reduce body weight and BMI compared to short term continuous energy restriction.
3. Further research is needed to assess the safety of intermittent fasting before recommendations can be made for the intervention to be implemented within clinical practice.
4. Given the limited certainty of evidence and the high risk of bias within existing studies, it is not yet possible to make universal recommendations regarding the implementation of intermittent fasting into clinical practice.
